# Astroglial and Neuronal Injury Markers (GFAP, UCHL-1, NfL, Tau, S100B) as Diagnostic and Prognostic Biomarkers in PTSD and Neurological Disorders

**DOI:** 10.3390/ijms27052374

**Published:** 2026-03-04

**Authors:** Ewa Alicja Ogłodek, Michal Bar

**Affiliations:** 1 Collegium Medicum, Jan Dlugosz University in Częstochowa, Waszyngtona 4/8 Street, 42-200 Częstochowa, Poland; 2 Department of Clinical Neuroscience, Faculty of Medicine, University of Ostrava, 17. Listopadu 1790, 708 52 Ostrava, Czech Republic

**Keywords:** astrocytes, blood–brain barrier, biomarkers, GFAP, UCHL-1, neurofilament light chain, neuroinflammation, neurodegeneration, neuronal injury, post-traumatic stress disorder, S100B, tau

## Abstract

Post-traumatic stress disorder (PTSD) is increasingly recognized as a neurobiological condition involving persistent neuroinflammation, glial dysfunction, and neuronal injury. Chronic stress induces dysregulation of the hypothalamic–pituitary–adrenal axis, mitochondrial impairment, oxidative stress, and activation of inflammatory signaling pathways, leading to blood–brain barrier (BBB) disruption and progressive neural damage. These processes are reflected in circulating biomarkers that provide insight into underlying molecular pathology. This article focuses on key astroglial and neuronal injury markers—glial fibrillary acidic protein (GFAP), ubiquitin C-terminal hydrolase L1 (UCHL-1), neurofilament light chain (NfL), tau protein, and S100B—as indicators of stress-related brain dysfunction in PTSD. GFAP and S100B reflect astrocyte activation and BBB permeability, while UCHL-1, NfL, and tau indicate neuronal injury, axonal degeneration, and cytoskeletal instability. Accumulating clinical and experimental evidence suggests that altered levels of these biomarkers are associated with symptom severity, cognitive impairment, and neuroinflammatory activity in PTSD, often overlapping with mechanisms observed in neurodegenerative disorders. This review summarizes the current understanding of the biological significance and clinical relevance of these biomarkers and highlights their potential utility for early diagnosis, disease monitoring, and risk stratification. The combined assessment of astroglial and neuronal markers may support a more precise, biologically grounded approach to PTSD and facilitate the development of personalized diagnostic and therapeutic strategies.

## 1. Introduction

Post-traumatic stress disorder (PTSD) develops following exposure to a traumatic event involving a threat to one’s life. According to the Diagnostic and Statistical Manual of Mental Disorders, Fifth Edition (DSM-5), PTSD is diagnosed following exposure to trauma that evokes fear, helplessness, or horror. Characteristic symptoms include intrusive memories (flashbacks), nightmares, intrusive trauma-related thoughts, physiological reactions to trauma-associated stimuli, and persistent avoidance of situations, people, or thoughts linked to the traumatic experience. These symptoms are accompanied by negative cognitive and affective changes—such as guilt, loss of trust, and emotional numbing—as well as persistent hyperarousal, including irritability, heightened vigilance, concentration difficulties, insomnia, and exaggerated startle responses. For a diagnosis to be established, symptoms must persist for more than one month, cause clinically significant distress or functional impairment, and not be attributable to psychoactive substances or other medical conditions.

Trauma may be direct (personal experience), indirect (witnessing traumatic events), or secondary, resulting from contact with individuals who have experienced violence or injury. Increasing evidence indicates that trauma may also arise in the context of neurological disease, particularly when associated with sudden loss of function, life-threatening circumstances, or irreversible bodily change. In this context, PTSD is no longer viewed solely as a psychiatric disorder but rather as a condition located at the intersection of psychopathology and neurobiology [1].

Importantly, the central hypothesis of this review assumes that PTSD should be conceptualized as a systemic neurobiological disorder rather than an exclusively psychiatric condition. Chronic stress exposure induces persistent molecular and cellular alterations—particularly neuroinflammation, oxidative stress, glial activation, and blood–brain barrier (BBB) dysfunction—which together constitute a measurable biological substrate of PTSD. This framework places PTSD within the spectrum of stress-related neurobiological disorders and provides a rationale for the use of objective biomarkers reflecting neuronal and glial injury.

### 1.1. PTSD in the Context of Neurological Diseases

Stroke, epilepsy, multiple sclerosis (MS), and neurodegenerative diseases such as Alzheimer’s disease (AD) and Parkinson’s disease (PD) may serve both as sources of trauma and as biological substrates for the development of post-traumatic symptoms. Stroke caused by ischemia or hemorrhage disrupts neuronal homeostasis and leads to energy failure, followed by glutamate excitotoxicity, oxidative stress, and chronic neuroinflammation. These processes result in neuronal degeneration accompanied by microglial and astroglial activation as well as blood–brain barrier disruption, contributing to persistent cognitive deficits and motor impairment [2]. For many patients, the sudden loss of neurological function itself represents a profound traumatic experience. At the molecular level, such injury is associated with reduced hippocampal neuroplasticity [3].

In epilepsy, particularly in severe or drug-resistant forms, post-traumatic stress may arise both from anticipatory anxiety related to future seizures and from the biological consequences of recurrent neuronal discharges. Repeated seizures induce oxidative stress, microglial activation, and ionic imbalance, leading to synaptic degradation and progressive neurodegenerative processes [4,5]. Neuroimaging studies further demonstrate structural and functional alterations within the amygdala and prefrontal cortex—regions critically involved in emotional regulation.

In multiple sclerosis, trauma results from the combined effects of neuroinflammation and psychological burden. Demyelination and axonal injury lead to sustained astrocytic and microglial activation, accompanied by impaired neuroplasticity [6]. The unpredictable disease course, recurrent relapses, and progressive neurological disability intensify feelings of helplessness and threat, thereby increasing vulnerability to post-traumatic symptoms [7].

Neurodegenerative diseases such as Alzheimer’s and Parkinson’s disease share overlapping mechanisms with PTSD, including oxidative stress, chronic inflammation, and neurodegeneration [8]. In Alzheimer’s disease, β-amyloid accumulation and tau hyperphosphorylation result in cytoskeletal destabilization and neurofibrillary tangle formation [9]. These changes promote excessive production of reactive oxygen species, microglial activation, and the release of proinflammatory cytokines, including interleukin-1β (IL-1β), interleukin-6 (IL-6), and tumor necrosis factor-α (TNF-α), accelerating synaptic loss and hippocampal dysfunction [10].

In Parkinson’s disease, progressive dopaminergic neuron degeneration, α-synuclein aggregation, and mitochondrial dysfunction result in sustained microglial activation and oxidative stress [11]. These processes contribute not only to motor impairment but also to affective and cognitive disturbances frequently observed in PTSD. Notably, these pathological mechanisms are driven by interconnected inflammatory, oxidative, and cellular stress pathways common to both neurodegenerative and stress-related disorders [12].

Persistent activation of mitogen-activated protein kinase/extracellular signal–regulated kinase (MAPK/ERK), protein kinase RNA-like endoplasmic reticulum kinase (PERK), and c-Jun N-terminal kinase (JNK) signaling pathways, together with dysregulation of the inositol-requiring enzyme 1–X-box binding protein 1 (IRE1–XBP1) axis, promotes endoplasmic reticulum stress and upregulation of proapoptotic mediators such as caspase-3 and Bax, ultimately leading to progressive neuronal injury [13].

### 1.2. Neurobiological Basis of PTSD: Oxidative Stress, Neuroinflammation, and BBB Dysfunction

The molecular pathology of PTSD involves dysregulation of the hypothalamic–pituitary–adrenal (HPA) axis, excessive secretion of cortisol and catecholamines, increased production of reactive oxygen species, and impairment of ubiquitin-dependent protein degradation pathways [14,15,16,17]. At the cellular level, PTSD is associated with activation of the NOD-like receptor family pyrin domain-containing 3 (NLRP3) inflammasome, elevated expression of proinflammatory cytokines (IL-1β, IL-6, TNF-α), mitochondrial dysfunction, and disturbed cellular energy metabolism [18,19,20].

Contemporary neurobiological models conceptualize PTSD as a disorder of maladaptive stress responses involving convergent neurochemical, inflammatory, and neurodegenerative mechanisms [21,22]. Recent integrative analyses emphasize the central role of glutamatergic dysregulation, mitochondrial impairment, neuroinflammation, and disrupted synaptic plasticity as core biological features of PTSD [23,24,25]. These findings further support the conceptual overlap between PTSD and neurodegenerative disorders, reinforcing the rationale for biomarker-based characterization of disease progression.

Oxidative stress promotes lipid peroxidation and DNA damage, whereas mitochondrial dysfunction leads to ATP depletion and cytochrome c release, thereby activating intrinsic apoptotic pathways [18]. Concurrent activation of matrix metalloproteinase-9 (MMP-9) and endothelial adhesion molecules increases blood–brain barrier permeability, facilitating the translocation of brain-derived proteins into the peripheral circulation [19].

### 1.3. Importance of Glial–Neuronal Biomarkers in PTSD and Neurological Disorders

Together, these biomarkers provide an integrated biological framework linking glial, neuronal, and vascular alterations in PTSD. Translational studies consistently demonstrate elevated concentrations of glial fibrillary acidic protein (GFAP), ubiquitin C-terminal hydrolase L1 (UCHL-1), neurofilament light chain (NfL), tau, and S100B in both neurodegenerative diseases and PTSD [20,21,22,23,24,25].

Glial fibrillary acidic protein and S100B primarily reflect the astrocytic activation and blood–brain barrier dysfunction described previously [20,22,23,24].

UCHL-1, a neuron-specific enzyme involved in ubiquitin-dependent proteostasis, reflects neuronal stress and impaired protein turnover [21].

These biomarkers do not represent isolated indicators but rather form a coherent neurobiological signature of PTSD. Their combined assessment enables multidimensional evaluation of neuroinflammation, axonal injury, glial activation, and BBB disruption, thereby redefining PTSD as a disorder with measurable neuropathological correlates.

From an early-detection perspective, GFAP and S100B may act as sensitive indicators of early astroglial activation and BBB dysfunction. UCHL-1—given its rapid release after neuronal stress—appears particularly promising as a potential early or preclinical marker that is detectable even before full PTSD diagnostic criteria are met. In contrast, NfL and tau are more consistent with cumulative axonal/cytoskeletal injury and chronic disease burden.

The selection of GFAP, UCHL-1, NfL, tau, and S100B was guided by three complementary criteria:

(i) Biological specificity for cellular compartments relevant to PTSD pathophysiology;
(ii) Mechanistic plausibility within stress-related molecular cascades;
(iii) Translational feasibility, as these proteins can be reliably quantified in peripheral blood and are routinely applied in clinical neuroscience.

Together, these biomarkers constitute a coherent panel reflecting astroglial activation, BBB dysfunction, neuronal stress, and axonal microdamage.

Integration of biomarker profiles with clinical symptomatology provides a robust framework for redefining PTSD as a neurobiological disorder with measurable structural and biochemical correlates, opening up new avenues for early diagnosis, risk stratification, and personalized therapeutic strategies [12,13,14,20,21,22,23,24,25].

## 2. Neurobiological Mechanisms of Brain Injury in PTSD

### 2.1. Neuroinflammation and Glial Reactivity

Neuroinflammatory and glial mechanisms play an important role in the pathophysiology of PTSD. There is growing evidence that chronic stress leads to the activation of microglia and astroglia [25,26]. Under physiological conditions, glial cells perform protective functions: they regulate energy metabolism and neurochemical homeostasis, contribute to the removal of abnormal proteins, and modulate synaptic plasticity. However, chronic exposure to traumatic stress can initiate inflammatory, oxidative, and neurodegenerative processes that perpetuate the neuronal and emotional dysfunctions observed in PTSD [27,28].

To illustrate the key neuron–microglia–astrocyte interactions, NLRP3 activation, and proinflammatory cytokine secretion in PTSD, a diagram of neuroinflammatory mechanisms is included.

Microglia are resident immune cells of the central nervous system with a predominantly yolk sac-derived developmental origin. However, their ontogeny and the contribution of peripheral myeloid populations under pathological conditions remain an area of ongoing investigation.

In response to stress, the HPA axis is activated, leading to the release of corticosteroids. As a result, microglia transition to a reactive M1 state in which they secrete proinflammatory cytokines such as IL-1β, IL-6, and TNF-α [29]. Prolonged activation of microglia of the M1 phenotype shifts the balance toward its neurotoxic form (M1) at the expense of the neuroprotective phenotype (M2), resulting in persistent inflammation and impaired neuronal function [29]. Postmortem studies and animal models of PTSD show increased expression of microglial markers such as ionized calcium-binding adapter molecule 1 (Iba-1), cluster of differentiation 11b (CD11b), and 18 kDa translocator protein (TSPO), particularly in the hippocampus, amygdala, and prefrontal cortex [30]. In these brain regions, a higher number of activated amoeboid-shaped microglial cells and increased production of reactive oxygen species (ROS) have been observed [31]. These changes are associated with memory impairment, excessive emotional reactivity, and anxiety—symptoms characteristic of PTSD [32].

Notably, glial reactivity within these regions appears to map onto partially distinct PTSD symptom dimensions. In the hippocampus, stress-related microglial activation and oxidative injury are most consistently linked to impaired contextual memory, reduced synaptic plasticity, and deficits in extinction learning. In the amygdala, inflammatory activation may preferentially support heightened threat detection, fear generalization, and exaggerated autonomic responses. In the prefrontal cortex, particularly within medial and dorsolateral subregions, glial-driven metabolic and inflammatory imbalance may contribute to weakened top–down inhibition of limbic circuits, thereby sustaining hyperarousal and intrusive symptomatology. This regional perspective is important for interpreting peripheral biomarker signals, as elevations in GFAP, S100B, NfL, or UCHL-1 may reflect different dominant circuit-level disturbances across PTSD phenotypes [3,7,26].

Astroglia, which constitute approximately 30–40% of brain cells, also undergo reactive changes in PTSD. Astrocytes are responsible for maintaining ionic balance, removing and recycling neurotransmitters (especially glutamate), forming the BBB, and regulating synaptic transmission [33]. Under chronic stress conditions, excess glutamate and corticosteroids lead to astrocyte activation and increased expression of GFAP [12]. Reactive astrocytes secrete larger amounts of cytokines (IL-6, IL-1β, TNF-α), chemokines (monocyte chemoattractant protein-1 (MCP-1), C-X-C motif chemokine ligand 10 (CXCL10)), and S100B protein, which in excess exerts proinflammatory and neurotoxic effects [33].

In addition to canonical neuroimmune pathways, accumulating evidence implicates the orexin (hypocretin) system as a modulatory node linking stress responses to hyperarousal and sleep–wake disturbances in PTSD. Orexinergic neurons in the lateral hypothalamus provide dense projections to the amygdala–hippocampus–prefrontal network and regulate arousal, vigilance, and autonomic tone. Dysregulated orexin signaling has been associated with fragmented sleep, heightened startle reactivity, and exaggerated stress responsivity, and may interact with glial activation through neuroendocrine amplification and sympathetic outflow. From a translational perspective, this suggests that orexin-related alterations could help contextualize biomarker profiles, particularly in PTSD subgroups characterized by prominent insomnia and hyperarousal [34,35].

Chronic activation of microglia and astroglia in PTSD disrupts the balance between neurons and glial cells, leading to mitochondrial dysfunction, increased oxidative stress, and BBB damage. Persistent glial reactivity may be one of the mechanisms underlying the chronic course of PTSD and its resistance to treatment [36].

### 2.2. Cytokine Pathways and NLRP3 Inflammasome Activation

One of the key mechanisms linking psychological stress to inflammatory processes in the brain is the activation of the NLRP3 inflammasome. This inflammasome acts as a molecular sensor that recognizes danger-associated molecular patterns (DAMPs) and pathogen-associated molecular patterns (PAMPs). It is activated in response to oxidative stress, mitochondrial damage, ion imbalance, and activation of Toll-like receptors (TLR2, TLR4). Its activation occurs in two stages: “priming,” which is nuclear factor kappa B (NF-κB)-dependent, and “triggering,” which involves an increase in ROS and calcium ion (Ca^2+^) influx [37,38].

In PTSD, elevated glucocorticosteroid levels promote increased expression of TLR4 receptors in microglia. Simultaneously, mitochondrial DNA fragmentation provides DAMP signals that stimulate NLRP3 inflammasomes [18].

This leads to recruitment of the apoptosis-associated speck-like protein containing a caspase recruitment domain (ASC) adaptor protein and activation of caspase-1, which converts the inactive forms of IL-1β and interleukin-18 (IL-18) into their active forms [39]. These cytokines enhance microglial activity, increase BBB permeability, and exacerbate neuronal apoptosis.

Studies have shown increased expression of NLRP3, apoptosis-associated speck-like protein containing an ASC, and caspase-1 in the hippocampus in PTSD models, which correlate with the severity of anxiety symptoms. Conversely, inflammasome inhibitors such as MCC950 (CP-456,773) reduce IL-1β and TNF-α levels, restore the balance between M1 and M2 phenotypes, and alleviate behavioral symptoms [15,16,17]. In related conditions, such as TBI, NLRP3 activation leads to excessive cytokine release, pyroptosis, and secondary neuronal damage, confirming a mechanism common to PTSD [14].

Oxidative stress and mitochondrial dysfunction, through activation of thioredoxin-interacting protein (TXNIP) and JNK and nuclear factor kappa B (NF-κB) activity, sustaining chronic neuroinflammation [19].

### 2.3. Disruption of Neuron-Astrocyte-Microglia Communication

Proper communication between neurons, astrocytes, and microglia is essential for maintaining brain homeostasis. In PTSD, this communication is disrupted in terms of both chemical signaling and the exchange of nutrients and energy. Astrocytes form a so-called tripartite synapse where they regulate glutamate and gamma-aminobutyric acid (GABA) concentrations in the synaptic space. Excess glutamate resulting from HPA axis hyperactivity causes toxic neuronal excitation (excitotoxicity). At the same time, reduced expression of the excitatory amino acid transporter 2 (EAAT2) in reactive astrocytes exacerbates this effect [29]. These cytokines further amplify neuroinflammatory signaling and contribute to synaptic dysfunction and impaired network plasticity. Interleukin-1β weakens long-term potentiation (LTP) in the hippocampus, contributing to memory deficits. TNF-α activates tumor necrosis factor receptor 1 (TNFR1), causing the loss of α-amino-3-hydroxy-5-methyl-4-isoxazolepropionic acid (AMPA) receptors and disruption of glutamatergic signaling [40]. Additionally, dysfunction of the C-X3-C motif chemokine ligand 1–C-X3-C motif chemokine receptor 1 (CX3CL1–CX3CR1) axis, which mediates neuron–microglia communication, increases microglial reactivity and ROS production [29]. The S100B protein, secreted by astrocytes, supports neuronal function but, in excess, activates the receptor for advanced glycation end products (RAGE), triggering the S100B–RAGE–NF-κB signaling pathway and promoting inflammation. As a result, sustained activation of microglia and astrocytes leads to increased levels of GFAP, S100B, UCHL-1, NfL, and tau in peripheral body fluids [21,41,42,43,44,45,46,47,48,49].

### 2.4. Clinical and Translational Significance

Understanding the neuroinflammatory and glial mechanisms in PTSD is essential for identifying new biomarkers and targeted therapies. The combination of glial-neuronal biomarkers (GFAP, UCHL-1, NfL, tau, S100B) with neuroimaging positron emission tomography targeting the 18 kDa translocator protein (PET-TSPO) allows for the assessment of the degree of inflammation and neurodegeneration. In experimental studies, modulation of microglia using minocycline, peroxisome proliferator-activated receptor gamma (PPAR-γ) agonists, or NLRP3 inhibitors (MCC950) has anti-inflammatory and neuroprotective effects [21,42,48]. The integration of cytokine biomarkers (IL-1β, IL-6, TNF-α, IL-18) with oxidative markers and glial-neuronal proteins may enable the development of new PTSD diagnostic panels [12,13,14].

## 3. Blood–Brain Barrier Dysfunction in PTSD

The blood–brain barrier plays a fundamental role in maintaining central nervous system homeostasis by regulating the exchange of molecules between the peripheral circulation and neural tissue. Under physiological conditions, this barrier limits the entry of potentially harmful substances while enabling controlled transport of nutrients and signaling molecules. In PTSD, increasing evidence suggests that BBB integrity may be compromised, contributing to neurovascular dysfunction and altered brain homeostasis [20,43,47].

Chronic activation of the HPA axis, a hallmark of prolonged stress exposure, results in sustained elevations of glucocorticoids and catecholamines. These neuroendocrine changes have been shown to adversely affect endothelial cells, leading to impaired tight junction organization and reduced barrier stability. In parallel, oxidative stress and metabolic disturbances further compromise endothelial function, thereby increasing BBB permeability. As a consequence, the normally restrictive properties of the BBB are weakened, allowing for enhanced exchange between the peripheral circulation and the central nervous system [49].

Stress-induced activation of microglia and astrocytes contributes to the release of proinflammatory mediators, including Il-1β, Il-6, and TNF-α. These cytokines have been shown to modulate endothelial permeability and promote neurovascular inflammation. In addition, oxidative and mitochondrial stress further aggravate endothelial injury by disrupting cellular energy homeostasis and enhancing reactive oxygen species production. Together, these mechanisms facilitate degradation of tight junction components and promote sustained BBB dysfunction, thereby linking psychological stress to the neuroinflammatory and vascular alterations observed in PTSD [50,51].

Increased BBB permeability enables the passage of brain-derived proteins into the systemic circulation, providing indirect indicators of central nervous system injury. Among the most frequently studied biomarkers are GFAP and S100B, which reflect astrocytic activation and BBB disruption, NfL, which is associated with axonal injury, UCHL-1, which reflects neuronal stress, and tau protein, which is linked to cytoskeletal instability. Notably, their presence in peripheral blood may precede overt clinical manifestations, suggesting potential utility for early detection [21,52].

Available evidence indicates that BBB dysfunction in PTSD may persist over time and correlate with symptom severity, particularly in the cognitive and affective domains. The assessment of BBB-related biomarkers therefore represents a promising approach for improving biological characterization of PTSD. While these markers are not yet suitable for routine clinical application, their combined evaluation may support differential diagnosis, aid in disease monitoring, and provide insight into treatment response. Further longitudinal studies are required to clarify their specificity, temporal dynamics, and clinical utility [53,54].

## 4. Neuronal Degeneration and Synaptic Dysfunction

Neuronal degeneration is an important component of the pathophysiology of PTSD and is one of the main manifestations of chronic stress. Persistent neuroinflammation and disturbances in protein homeostasis (proteostasis) lead to progressive neuronal damage, including axonal degeneration, cytoskeletal destabilization, and synaptic dysfunction [55,56,57,58]. As a result, neuronal plasticity is impaired, and the brain’s adaptive mechanisms are disrupted. These phenomena are relevant not only in PTSD but also in other trauma-related neurological conditions, such as TBI, chronic traumatic encephalopathy (CTE), and neurodegenerative diseases such as Alzheimer’s disease [9,11,23,59].

In recent years, three biomarkers of neuronal damage have gained particular importance: tau protein, UCHL-1, and neurofilament light chain. Their interrelationships reflect successive stages of the neurodegenerative process, from early cytoskeletal disturbances to advanced axonal loss and synaptic dysfunction. Tau protein, a microtubule stabilizer within the neuronal cytoskeleton, plays an essential role in maintaining axonal transport and neuronal integrity [21,22].

Importantly, tau alterations observed in PTSD primarily reflect stress-induced phosphorylation and functional destabilization of microtubules rather than the progressive tau aggregation characteristic of classical tauopathies such as Alzheimer’s disease or chronic traumatic encephalopathy [9,45,47,48,49].

Under conditions of oxidative stress, kinases such as glycogen synthase kinase 3 beta (GSK-3β) and cyclin-dependent kinase 5 (CDK5) become overactivated, leading to hyperphosphorylation of tau [49].

The altered protein loses its ability to bind microtubules, thereby destabilizing microtubules and impairing cytoskeletal integrity. In PTSD and other neuroinflammatory diseases, increased concentrations of phosphorylated tau (p-tau) correlate with microglial activation, BBB dysfunction, and neuroinflammatory processes in the hippocampus and prefrontal cortex. Pathological forms of tau have also been shown to spread between neurons in a prion-like manner, exacerbating neural network damage and disrupting memory and emotional processing [12,51].

The second key biomarker is UCHL-1, a deubiquitinating enzyme involved in maintaining proteostatic balance through ubiquitin recycling and regulation of the ubiquitin–proteasome pathway. Ubiquitin C-terminal hydrolase L1 dysfunction, resulting from oxidative modifications or transcriptional abnormalities, leads to the accumulation of abnormal proteins, endoplasmic stress, and caspase activation, thereby exacerbating neuronal apoptosis. Elevated serum UCHL-1 levels in patients with PTSD, as well as in TBI, confirm its importance as a marker of neuronal and synaptic damage. Loss of UCHL-1 function is also associated with impaired synaptic plasticity, reflected in memory and cognitive deficits. In animal models, chronic stress leads to decreased UCHL-1 expression in the hippocampus, resulting in impaired spatial learning and loss of long-term synaptic potentiation. Disturbances in synaptic protein turnover are among the mechanisms perpetuating memory and emotional dysfunction [21,40,52].

Neurofilament light chain, on the other hand, is a sensitive marker of axonal damage associated with the loss of neuronal cytoskeletal integrity. Under conditions of oxidative stress or protease activation, NfL fragments enter the cerebrospinal fluid and blood, providing an objective indicator of neuronal degeneration. Individuals with PTSD show elevated NfL levels, which may persist for many years after trauma exposure, indicating chronic axonal microdamage. Similar changes are observed in TBI and neurodegenerative diseases such as Alzheimer’s disease, confirming a shared stress-dependent mechanism of neuronal injury [22,23,44,52].

The mechanisms involving tau, UCHL-1, and NfL are interrelated. Dysfunction of the UCHL-1-dependent ubiquitin–proteasome pathway impairs the degradation of pathologically phosphorylated tau, resulting in microtubule destabilization [40,45,60,61,62,63,64]. Cytoskeletal damage increases neuronal susceptibility to oxidative stress, and released NfL fragments can activate microglia and astroglia, amplifying the neuroinflammatory cascade. This creates a cycle of damage where oxidative stress, loss of proteostasis, and neuroinflammatory activation mutually exacerbate neuronal degeneration [22,45,65,66,67,68,69].

These disorders are linked to neuroplasticity. In PTSD, there is a reduction in dendritic spine density, decreased expression of plasticity-related genes (brain-derived neurotrophic factor (BDNF), synapsin-1, postsynaptic density protein 95 (PSD-95)), and a reduction in the volume of pyramidal neurons in the hippocampus [46,70,71].

The loss of neural network flexibility results in contextual memory deficits, amygdala hyperactivity, and emotional regulation disturbances—key features of the clinical profile of PTSD. Similar phenomena have been observed in chronic post-traumatic encephalopathies, supporting the existence of common mechanisms of neuroinflammatory dysregulation [72,73,74].

In light of these findings, comprehensive assessment of neuronal biomarkers such as tau, UCHL-1, and NfL is a promising diagnostic tool in PTSD and trauma-related neurological diseases. The combination of biomarker analysis with neuroimaging methods such as magnetic resonance imaging (MRI), diffusion tensor imaging (DTI), and positron emission tomography (PET) and neuropsychological testing may enable early detection of damage, monitoring of disease progression, and evaluation of neuroprotective therapy effectiveness. Such an approach, consistent with the principles of precision medicine, may, in the future, contribute to the development of personalized treatment strategies for PTSD and related disorders in which neuronal degeneration is a common pathophysiological link [75,76].

### 4.1. Astroglial and Neuronal Biomarkers of Injury

In recent years, increasing attention has been paid to astroglial and neuronal biomarkers as important indicators of central nervous system damage. Unlike neuroimaging methods, which show structural or functional changes, these biomarkers reflect molecular processes occurring in the brain, such as glial activation, oxidative stress, axonal damage, disturbances in neuron-astrocyte communication, and BBB dysfunction.

In the context of PTSD, this is particularly relevant because these biomarkers allow for the detection of subtle neuronal and glial damage that may remain undetectable using standard neuroimaging techniques such as MRI or CT.

The most extensively studied biomarkers include GFAP, UCHL-1, NfL, tau, and S100B, which together form a molecular signature reflecting both astroglial activation and neuronal injury [22,23,40,43,45,53]. Importantly, accumulating clinical and experimental evidence indicates that alterations in these markers are not limited to acute brain trauma but are also observed in chronic stress-related and neuropsychiatric conditions, including PTSD, depression, and anxiety disorders [77,78].

Recent studies have demonstrated that these biomarkers undergo measurable changes in a wide range of neuropsychiatric disorders, supporting their relevance beyond classical neurodegenerative or traumatic conditions [79].

Elevated GFAP and S100B levels have been associated with astroglial activation, neuroinflammatory processes, and impaired barrier integrity in stress-related disorders, whereas increased NfL concentrations reflect the axonal damage and neuroprogression observed in affective and trauma-related psychopathology [22].

Moreover, growing evidence indicates that UCHL-1 and tau, although initially validated in traumatic brain injury, are also altered in chronic stress exposure and psychiatric disorders, where they are linked to synaptic dysfunction, impaired proteostasis, and neurodegenerative-like processes. These findings provide a mechanistic bridge between structural and molecular brain pathology and the measurable peripheral biomarkers observed in neuropsychiatric populations, reinforcing their translational value in PTSD and related conditions [80,81].

For clarity and translational relevance, the key astroglial and neuronal biomarkers associated with PTSD are summarized in Table 1. The table integrates cellular origin, primary biological function, pathophysiological relevance, and potential clinical utility, highlighting mechanisms linking PTSD with neuroinflammation, BBB dysfunction, and neurodegenerative-like processes.

Collectively, these biomarkers provide a multidimensional molecular profile of PTSD, capturing astroglial activation, neuronal injury, axonal degeneration, and blood–brain barrier dysfunction. This integrated biomarker profile supports the growing concept of PTSD as a stress-related neurobiological disorder rather than a purely psychological condition. Their combined assessment may therefore serve as a foundation for biomarker-guided precision diagnostics and treatment monitoring [81,82].

Glial fibrillary acidic protein is a structural intermediate filament protein expressed predominantly in astrocytes and represents one of the most widely used markers of astroglial activation [40,44,45,52]. Elevated GFAP concentrations have been reported in traumatic brain injury, stroke, neurodegenerative disorders, and stress-related conditions, including PTSD.

In PTSD, increased GFAP levels are thought to reflect astrocytic reactivity, neuroinflammation, and BBB disruption driven by chronic HPA-axis activation and oxidative stress. Clinical studies demonstrate that elevated circulating GFAP can be detected even in the absence of overt structural brain damage, suggesting its usefulness as a sensitive marker of stress-related astroglial dysfunction. Importantly, GFAP—together with UCHL-1—constitutes the basis of the FDA-approved Brain Trauma Indicator, underscoring its translational relevance in detecting subtle brain injuries [52,66].

Ubiquitin C-terminal hydrolase L1 is a neuron-specific enzyme involved in ubiquitin recycling and maintenance of protein homeostasis. Although originally validated as a biomarker of traumatic brain injury, increasing evidence indicates its relevance in stress-related neuropsychiatric disorders. Clinical studies in PTSD patients have demonstrated elevated circulating UCHL-1 levels, which correlate with symptom severity, cognitive impairment, and sleep disturbances. Chronic stress appears to impair UCHL-1 function through oxidative modification, leading to protein aggregation, synaptic dysfunction, and activation of endoplasmic reticulum stress pathways. These mechanisms suggest that UCHL-1 may represent an early indicator of stress-induced neuronal dysfunction and neurodegenerative-like processes [21,24,66].

Neurofilament light chain is a structural component of axons and a well-established marker of axonal injury. Upon neuronal damage, NfL is released into cerebrospinal fluid and peripheral blood, making it one of the most sensitive indicators of neurodegeneration [45].

In PTSD, elevated NfL levels have been associated with chronic axonal microdamage driven by oxidative stress, neuroinflammation, and dysregulation of the HPA axis. Clinical and neuroimaging studies demonstrate significant correlations between increased NfL levels and white matter microstructural abnormalities, cognitive impairment, and disease chronicity in PTSD patients, further supporting its utility as a biomarker of long-term neuronal damage [22,23,45].

Tau plays a key role in microtubule stabilization and axonal transport. Under conditions of oxidative stress and kinase overactivation, tau undergoes pathological hyperphosphorylation, leading to cytoskeletal destabilization and synaptic dysfunction.

In PTSD, elevated levels of phosphorylated tau have been linked to impairments in memory, emotional regulation, and executive functioning. Experimental and clinical data suggest that chronic psychological stress may induce tau-related neurodegenerative processes similar to those observed in Alzheimer’s disease and chronic traumatic encephalopathy, particularly in the hippocampus and prefrontal cortex [83,84,85].

S100B is a calcium-binding protein predominantly produced by astrocytes. At physiological concentrations, it exerts neurotrophic effects; however, at elevated levels, it promotes neuroinflammation via RAGE signaling and reflects BBB dysfunction.

In PTSD, increased circulating S100B levels are interpreted as indicators of astroglial activation and BBB permeability rather than peripheral release. Clinical studies have demonstrated associations between elevated S100B, depressive symptoms, sleep disturbances, and markers of oxidative stress. Experimental models further show increased S100B expression in the hippocampus and prefrontal cortex, accompanied by reduced expression of synaptic plasticity markers such as BDNF and synaptophysin [24,40,43].

Together, these markers support a translational framework for interpreting stress-related glial and neuronal injury in PTSD [86,87].

Future studies integrating these biomarkers with inflammatory mediators (e.g., IL-1β, IL-6, TNF-α) and neurotrophic factors (BDNF, insulin-like growth factor 1 (IGF-1)) may enable the development of multimodal diagnostic panels, facilitating personalized treatment strategies and objective monitoring of therapeutic response in PTSD and related disorders [41].

### 4.2. Comparative Analysis: PTSD vs. Neurological Disorders

Post-traumatic stress disorder is increasingly being viewed from the perspective of neurobiological disorders, in which neuronal and glial dysfunctions typical of neurological diseases occur [63].

This section provides a clinically oriented synthesis of biomarker dynamics across PTSD, traumatic brain injury, and neurodegenerative disorders. Table 2 and Table 3 summarize differences in biomarker patterns, highlighting whether observed changes reflect transient injury responses or persistent stress-related neurobiological alterations.

Accordingly, Table 2 summarizes preclinical–clinical convergence for each marker, whereas Table 3 highlights disorder-specific differences in biomarker trajectories and reversibility, supporting the concept of PTSD as a stress-related neurobiological condition with partially overlapping mechanisms [20,21,22,23,24,25].

A common mechanism in these disorders is the activation of neuroinflammatory processes and damage to astrocytes and neurons, which is reflected in changes in biomarker levels such as GFAP, UCHL-1, NfL, tau, and S100B levels [23,24,52]. In PTSD, elevated levels of GFAP and S100B are observed, indicating astroglial activation and increased BBB permeability. At the same time, increases in UCHL-1 and NfL suggest disturbances in neuronal and axonal integrity, similar to those seen in mild traumatic brain injury (mTBI) [52,66]. These microstructural changes indicate the impact of chronic stress and activation of the HPA axis, leading to increased glucocorticoids and oxidative stress in limbic neurons.

To enhance the translational value of this review, Table 2 provides a comparative overview of astroglial and neuronal biomarkers across preclinical and clinical studies of PTSD, traumatic brain injury, and neurodegenerative disorders. This comparison highlights convergent and divergent biomarker profiles, thereby supporting the concept of PTSD as a stress-related neurobiological condition with partially overlapping molecular mechanisms.

To better contextualize PTSD within the spectrum of stress-related and neurodegenerative disorders, Table 3 compares key astroglial and neuronal biomarker profiles across PTSD, TBI, and AD, highlighting shared and disorder-specific mechanisms.

Unlike neurodegenerative diseases such as AD or chronic traumatic encephalopathy (CTE), the biomarker profile in PTSD suggests a role for glial dysfunction with relatively lower accumulation of tau and phospho-tau proteins. In Alzheimer’s disease, increased p-tau and β-amyloid lead to neurodegeneration, whereas in PTSD, moderate increases in tau correlate with cognitive and sleep disturbances but not with neuronal loss [65]. Ubiquitin C-terminal hydrolase L1, a cytoplasmic neuronal enzyme, shows persistent elevation in individuals with chronic PTSD, indicating ongoing activation of protein degradation mechanisms and proteostatic stress. Meanwhile, NfL, a marker of axonal fiber damage, is associated with the severity of avoidance and hyperarousal symptoms, which may reflect dysfunction in connections between the amygdala, prefrontal cortex, and hippocampus. In TBI, GFAP and UCHL-1 levels rise sharply after injury and correlate with the severity of damage, whereas in PTSD, these changes are less pronounced but persist chronically [84]. This suggests that PTSD exists on a continuum between psychological stress and neurobiological disease in which glial neuroinflammatory processes and synaptic dysfunction constitute a key link in its pathophysiology. In summary, a comparison of biomarker profiles shows that PTSD is characterized by a pattern associated with chronic increases in GFAP, UCHL-1, and S100B, along with moderate increases in NfL and tau—but without features of progressive neurodegeneration [44]. This points to the potential reversibility of neuronal and glial disturbances if appropriate therapeutic intervention is provided.

## 5. Diagnostic and Prognostic Value of Combined Biomarker Panels

The use of single biomarkers in the diagnosis of post-traumatic stress disorder (PTSD) remains limited due to insufficient specificity and sensitivity when applied in isolation. Consequently, increasing attention has been directed toward multi-marker panels integrating astroglial, neuronal, inflammatory, and oxidative stress indicators in order to provide a more comprehensive and clinically meaningful characterization of PTSD. Such integrative approaches more accurately reflect the multifactorial nature of the disorder, which involves neuroinflammation, synaptic dysfunction, BBB impairment, and dysregulation of stress-response systems.

From a clinical standpoint, multi-marker panels are most informative when they are anchored to symptom domains, chronicity, and functional outcomes rather than presented as isolated laboratory readouts. Therefore, in this section we emphasize that astroglial markers (GFAP, S100B) are most plausibly linked to BBB-related and sleep/affective phenotypes, whereas neuronal and axonal markers (UCHL-1, NfL, tau) map more closely onto cognitive burden, disease chronicity, and network-level dysconnectivity. This framework clarifies why the proposed panels in Table 4 integrate astroglial and neuronal components and supports their use for risk stratification and treatment monitoring in PTSD and related neuropsychiatric conditions [20,21,22,23,24,25,85].

Table 4 summarizes selected biomarker panels that have been proposed for diagnostic and prognostic purposes in PTSD. These panels combine molecular indicators of astrocyte activation, neuronal injury, and systemic inflammation with psychometric measures, thereby improving the interpretability of biological findings in a clinical context.

Importantly, most available human studies report *correlational* associations between peripheral biomarker concentrations and PTSD symptom severity. These findings should not be interpreted as evidence of direct causality. Biomarker alterations may reflect downstream epiphenomena, compensatory biological responses, or shared effects of confounding factors such as sleep disturbance, cardiometabolic burden, chronic inflammation, or medication exposure.

At present, there is insufficient evidence to conclude whether observed changes in GFAP, S100B, NfL, tau, or UCHL-1 represent predisposing vulnerability markers, consequences of trauma exposure, or correlates of symptom severity. Establishing causal directionality requires longitudinal study designs, repeated biomarker measurements, mediation analyses, and integration with neuroimaging and experimental paradigms.

Levels of biomarkers such as GFAP, UCHL-1, NfL, tau, and S100B show significant associations with clinical symptom severity assessed using validated psychometric instruments, including Clinician-Administered PTSD Scale for DSM-5 (CAPS-5), Pittsburgh Sleep Quality Index (PSQI), Beck Depression Inventory, and the Courtauld Emotional Control Scale (CECS). Elevated GFAP concentrations have been linked to more severe re-experiencing symptoms and impaired emotional regulation, whereas increased S100B levels correlate with sleep disturbances, supporting the role of astrocytic dysfunction in circadian rhythm disruption. UCHL-1 has been associated with depressive symptoms and anhedonia, while elevated NfL and tau reflect cognitive impairment and altered cortico–subcortical connectivity.

Proteomic analyses and meta-analytic data indicate that combinations of GFAP and UCHL-1 provide particularly high sensitivity for detecting stress-related brain injury, and that the addition of S100B and tau improves discrimination between PTSD and related psychiatric conditions such as depression or anxiety disorders. Integration of biomarker data with psychometric measures further enhances the predictive value of diagnostic models. Machine learning approaches, including Random Forest, Support Vector Machines, and Deep Neural Networks, have demonstrated high classification accuracy when biochemical, neuroimaging, and clinical variables are combined, with reported accuracies exceeding 85% in some studies.

Despite these promising findings, several important limitations currently restrict the clinical implementation of biomarker-based panels in PTSD. One major challenge is the substantial inter-assay and inter-laboratory variability observed for key biomarkers such as GFAP, S100B, and NfL, which are measured using different analytical platforms with limited methodological standardization. In addition, pre-analytical factors—including sample handling, circadian variation, comorbid medical conditions, and medication use—can significantly influence biomarker concentrations and complicate cross-study comparisons.

Another major limitation is the lack of universally accepted cutoff values defining pathological biomarker ranges specific to PTSD. Most available studies rely on group-level comparisons rather than clinically actionable thresholds, and biomarker distributions frequently overlap with those observed in depression, anxiety disorders, or mild traumatic brain injury. This limits their diagnostic specificity and underscores the need for large, multicenter validation studies.

Moreover, most existing studies are cross-sectional in design, which restricts causal inference. It therefore remains unclear whether observed biomarker alterations represent predisposing vulnerability factors, downstream consequences of chronic stress exposure, or correlates of symptom severity. Longitudinal studies incorporating repeated biomarker measurements, neuroimaging, and clinical follow-up are essential to clarify temporal relationships and determine true prognostic value.

Taken together, these observations indicate that biomarker panels in PTSD should currently be regarded as adjunctive research tools rather than stand-alone diagnostic tests. Their translation into routine clinical practice will require methodological harmonization, longitudinal validation, and integration with psychometric and neuroimaging data. Nonetheless, the growing body of evidence supports the concept that combined biomarker approaches provide valuable insight into the neurobiological underpinnings of PTSD and represent a promising step toward precision psychiatry.

## 6. Clinical and Translational Implications

Although the strongest validation of GFAP, UCHL-1, NfL, tau, and S100B originates from neurological and traumatic brain injury cohorts, accumulating evidence indicates their relevance in neuropsychiatric research, particularly in stress-related conditions. In PTSD, biomarker alterations are increasingly interpreted as peripheral correlates of central processes—glial reactivity, neuroinflammation, BBB permeability, axonal microdamage, and proteostasis stress—rather than as markers of overt macroscopic lesions. Experimental stress paradigms similarly demonstrate that chronic stress and neuroinflammatory priming can induce astroglial activation, oxidative injury, and synaptic dysfunction, providing mechanistic support for the observed clinical associations and strengthening the translational bridge between brain pathology and circulating markers [12,20,21,22,23,24,25,51,85].

An increasing number of studies suggest that astroglial and neuronal biomarkers—such as GFAP, UCHL-1, NfL, tau, and S100B—can serve not only as diagnostic tools but also as indicators of treatment response and clinical prognosis in PTSD patients [73].

Changes in their circulating levels during therapy reflect processes related to neuroplasticity, neuroinflammatory resolution, and restoration of neural homeostasis rather than direct reversal of structural damage.

**Figure 1:**
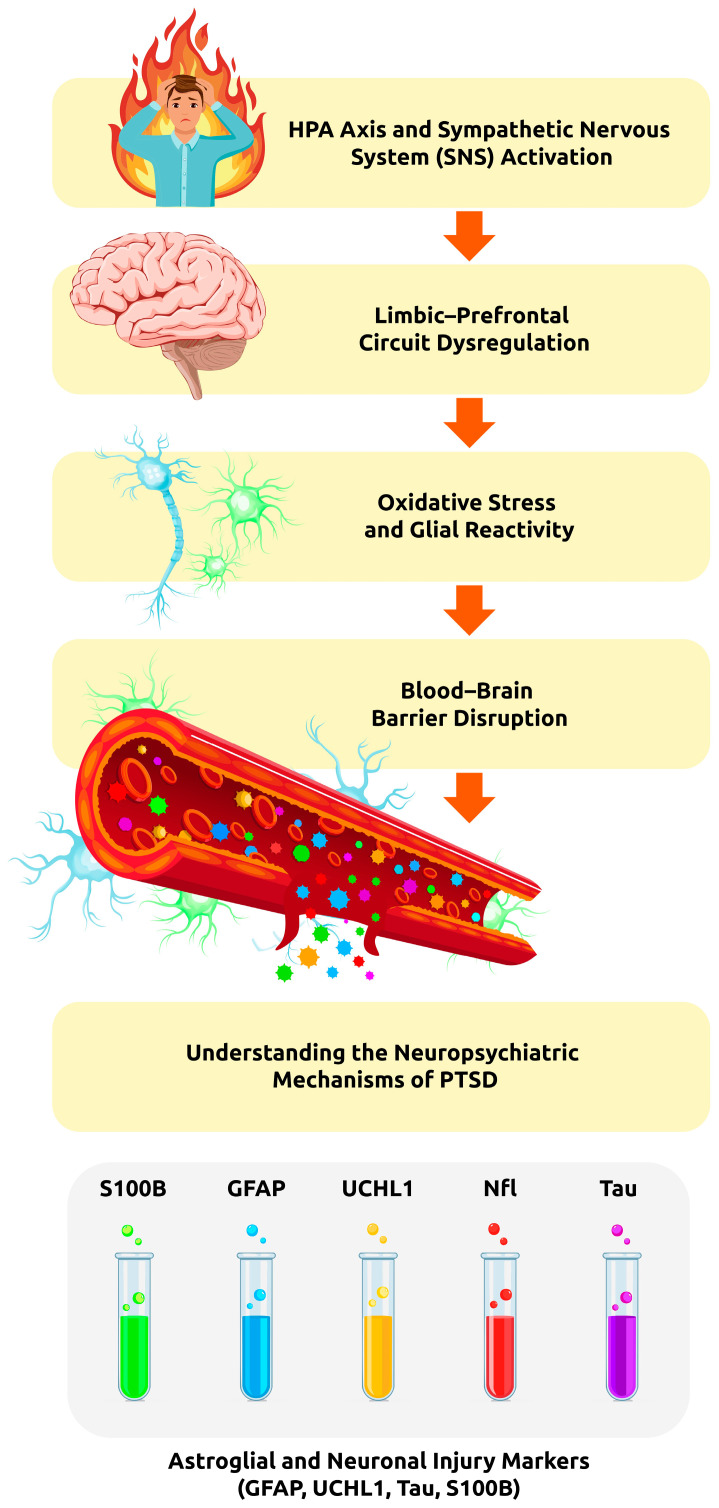
**Neurobiological cascade linking traumatic stress to circulating biomarkers in PTSD.** Traumatic stress activates the HPA axis and sympathetic nervous system, leading to limbic–prefrontal dysregulation, oxidative stress, and glial activation. Subsequent blood–brain barrier disruption enables the release of astroglial and neuronal injury markers into peripheral blood.

To improve conceptual clarity and translational interpretability, Figure 1 illustrates the proposed stress-to-biomarker cascade in PTSD, integrating neuroendocrine activation, glial reactivity, BBB dysfunction, and the subsequent release of astroglial and neuronal injury markers into peripheral circulation. This schematic framework highlights the sequential and interconnected nature of these processes and emphasizes that circulating biomarkers represent indirect readouts of central nervous system pathology rather than direct measures of tissue damage.

Translational studies have demonstrated that effective pharmacotherapy—such as selective serotonin reuptake inhibitors (SSRIs), serotonin–norepinephrine reuptake inhibitors (SNRIs), and N-methyl-D-aspartate (NMDA) receptor antagonists—may lead to reductions in GFAP and S100B levels, reflecting attenuation of astrocytic activation and partial stabilization of BBB integrity [74]. Concurrently, decreases in UCHL-1 and NfL have been associated with improved cognitive performance and reduced avoidance symptoms, suggesting partial restoration of axonal integrity and network function [75].

Psychotherapeutic interventions, including cognitive behavioral therapy (CBT) and eye movement desensitization and reprocessing (EMDR), also modulate limbic system activity and influence biomarker expression. Clinical improvement assessed using CAPS-5 and CECS has been shown to correlate with normalization of GFAP and UCHL-1 concentrations after approximately 12 weeks of treatment, supporting their potential utility as biological indicators of therapeutic response.

Importantly, reported reductions in biomarker levels during pharmacological or psychotherapeutic interventions should be interpreted with caution. Although temporal co-variation between biomarker normalization and symptom improvement is clinically encouraging, it does not demonstrate a causal relationship between biomarker modulation and therapeutic response. Observed changes may reflect secondary effects of treatment—such as reduced systemic inflammation, improved sleep quality, altered metabolic state, or decreased stress exposure—rather than direct reversal of central nervous system injury.

Therefore, biomarker dynamics should not be interpreted as mechanistic mediators of treatment efficacy without additional evidence. Prospective studies incorporating predefined biomarker endpoints, longitudinal sampling, and multimodal neuroimaging are required to determine whether observed biomarker changes truly reflect neurobiological recovery or represent epiphenomenal correlates of symptom improvement.

Regular biomarker monitoring may nevertheless provide clinically useful information regarding long-term outcomes. Persistently elevated GFAP and NfL levels after treatment may indicate ongoing neurobiological vulnerability or risk of progression toward neurodegenerative processes [40,44,45]. In contrast, reductions in UCHL-1 and phosphorylated tau (p-tau) concentrations may reflect improved proteostasis and stabilization of cytoskeletal dynamics, as observed in studies investigating neuroprotective strategies such as BDNF- or IGF-1-mediated signaling modulation [76].

The integration of biochemical markers with neuroimaging represents a key step toward translational PTSD models. Increased GFAP and NfL levels have been associated with reduced fractional anisotropy in the corpus callosum and weakened prefrontal–amygdala connectivity [22,45], while elevated S100B correlates with altered hippocampal glucose metabolism assessed using positron emission tomography [24,40,78]. Such multimodal approaches enable the construction of “post-traumatic stress biomaps”, facilitating the identification of PTSD subtypes characterized by predominant neuroinflammatory, metabolic, or neurodegenerative profiles and supporting personalized treatment strategies [79].

### 6.1. Study Design

This manuscript is a narrative integrative review evaluating astroglial and neuronal injury biomarkers—GFAP, UCHL-1, NfL, tau (including p-tau where available), and S100B—as diagnostic and prognostic indicators in PTSD and selected neurological disorders with overlapping mechanisms (neuroinflammation, BBB dysfunction, neurodegenerative-like pathways).

### 6.2. Data Sources and Search Strategy

PubMed/MEDLINE, Scopus, and Web of Science were searched for articles published between January 2000 and June 2025. Search terms combined PTSD/trauma-related keywords with biomarker names and mechanistic terms (e.g., *neuroinflammation*, *microglia/astrocytes*, *blood–brain barrier*, *oxidative stress*, *mitochondrial dysfunction*). Reference lists of key papers were also manually screened.

### 6.3. Eligibility Criteria

Inclusion criteria: peer-reviewed original studies and high-quality reviews reporting data for ≥1 target biomarker (GFAP, UCHL-1, NfL, tau/p-tau, S100B) in PTSD and/or relevant neurological cohorts (e.g., TBI, AD, PD, MS, stroke), including biomarker associations with symptoms, cognition, sleep, neuroimaging, or inflammatory/BBB markers.

Exclusion criteria: case reports, conference abstracts without full text, editorials/letters without data, non-relevant studies, and papers lacking key methodological details (sample type, assay, population characterization).

### 6.4. Study Selection and Data Extraction

Records were screened by title/abstract and then full text. Extracted variables included: study design; cohort characteristics (diagnosis, trauma type/chronicity, comorbidities); biomarker matrix (serum/plasma/CSF) and assay platform; direction of biomarker change; and reported clinical or mechanistic correlates (symptom severity, cognition, sleep, BBB/neuroinflammation indices).

### 6.5. Evidence Synthesis

Due to heterogeneity in assays, sampling timepoints, and outcome measures, findings were synthesized qualitatively (no meta-analysis). Evidence was integrated into mechanistic domains: glial reactivity/neuroinflammation, BBB dysfunction, neuronal/axonal injury, and clinical utility of single markers vs. multi-marker panels, with contextual comparison to neurological disorders to interpret acute vs. chronic biomarker trajectories.

## 7. Conclusions and Future Directions

Despite progress, research on PTSD biomarkers still needs to confirm the stability and causality of the observed changes. Most of the studies to date have been cross-sectional, making it difficult to determine whether changes in GFAP, UCHL-1, NfL, tau, and S100B levels are the cause or effect of PTSD symptoms. Longitudinal studies will allow us to determine the trajectories of biomarker changes and their relationship to remission, recurrence, or chronicity of the disorder. The integration of data from proteomics, metabolomics, neuroimaging, and neural network analysis will enable a more complete understanding of the mechanisms of PTSD. Combining biomarkers (GFAP, UCHL-1), metabolic markers (N-acetylaspartate (NAA), gamma-aminobutyric acid (GABA), reduced glutathione (GSH)), and functional magnetic resonance imaging (fMRI) or DTI data may reveal new pathophysiological pathways. Advances in multi-omics technology will enable the development of predictive models identifying PTSD subtypes with different risks of neurodegeneration and different therapeutic responses [78,79]. In the future, it will be possible to create individual biological profiles of PTSD based on panels combining GFAP, UCHL-1, NfL, tau, S100B, and cytokines (IL-6, IL-18). This approach will enable more accurate differentiation of PTSD from depressive disorders or post-traumatic encephalopathies, supporting the development of personalized medicine.

Although classified as a mental disorder, PTSD leads to permanent neurobiological changes resembling the processes observed in neurodegenerative diseases. These include microglial activation, astrocyte damage, and neuronal imbalance. The biomarkers GFAP, UCHL-1, NfL, tau, and S100B are sensitive indicators of these processes, linking the neurobiology of stress to neuronal pathology. Their integration with neuroimaging methods may enable earlier diagnosis of individuals at risk of chronic PTSD and support the development of personalized therapeutic strategies. The use of neuron-astroglial biomarkers in clinical practice opens a new era in biological psychiatry in which the diagnosis and assessment of post-traumatic stress is based not only on symptoms, but also on objective molecular and functional indicators of the brain.

## Figures and Tables

**Table 1:** Astroglial and neuronal biomarkers of brain injury in PTSD: cellular origin, biological significance, and clinical relevance.

Biomarker	Cellular Origin	Physiological Role	Pathophysiological Significance in PTSD	Clinical and Translational Relevance
GFAP	Astrocytes	Structural stability of astrocytic cytoskeleton; maintenance of BBB integrity	Reflects astroglial reactivity, neuroinflammation, and BBB disruption induced by chronic stress and HPA axis dysregulation	Marker of astrocyte activation and BBB damage; useful for monitoring stress-related brain injury and treatment response
UCHL-1	Neurons (cytoplasmic)	Ubiquitin recycling; regulation of proteostasis and synaptic protein turnover	Indicates neuronal stress, impaired protein degradation, synaptic dysfunction, and early neurodegenerative-like processes	Sensitive marker of neuronal and synaptic injury; component of FDA-approved Brain Trauma Indicator
NfL	Axons	Structural integrity of axons; impulse conduction	Reflects chronic axonal microdamage driven by oxidative stress and neuroinflammation	Marker of long-term axonal injury; correlates with cognitive impairment and white matter alterations
Tau	Neurons (axonal cytoskeleton)	Microtubule stabilization and axonal transport	Hyperphosphorylation under stress leads to cytoskeletal destabilization and synaptic dysfunction	Indicator of microtubular pathology and cognitive deficits; links PTSD with neurodegenerative pathways
S100B	Astrocytes	Regulation of neuronal growth and calcium homeostasis	At high levels, promotes inflammation via RAGE signaling and reflects BBB permeability	Marker of astrocyte activation and BBB leakage; correlates with affective and sleep disturbances

Table 1 summarizes key astroglial and neuronal biomarkers of brain injury in PTSD, detailing their cellular origin, physiological roles, pathophysiological significance, and clinical or translational relevance. The table highlights mechanisms linking chronic stress with astroglial activation, neuronal and axonal injury, blood–brain barrier dysfunction, and neurodegenerative-like processes. Abbreviations: GFAP, glial fibrillary acidic protein; BBB, blood–brain barrier; UCHL-1, ubiquitin C-terminal hydrolase L1; NfL, neurofilament light chain; FDA, U.S. Food and Drug Administration; RAGE, receptor for advanced glycation end products; PTSD, post-traumatic stress disorder; HPA, hypothalamic–pituitary–adrenal.

**Table 2:** Comparison of astroglial and neuronal biomarkers in preclinical and clinical PTSD-related studies.

Biomarker	Cellular Origin	Preclinical Evidence (Animal Models)	Clinical Evidence (Human Studies)	Translational Relevance
GFAP	Astrocytes	Increased GFAP expression in hippocampus and prefrontal cortex after chronic stress; reflects astrocyte reactivity and BBB dysfunction	Elevated serum GFAP in PTSD and stress-related disorders; correlates with symptom severity and neuroimaging markers of BBB disruption	Marker of astroglial activation and BBB integrity; useful for monitoring neuroinflammation
S100B	Astrocytes	Increased expression in stress-exposed rodents; associated with neuroinflammation and glial activation	Elevated plasma S100B in PTSD; correlates with sleep disturbance, affective symptoms, and BBB permeability	Indicator of astrocyte activation and stress-related BBB leakage
UCHL-1	Neurons (cytoplasmic)	Increased expression after stress-induced neuronal injury; linked to impaired proteostasis and synaptic dysfunction	Elevated serum levels in PTSD and TBI; associated with cognitive impairment and emotional dysregulation	Sensitive marker of neuronal stress and early neurodegeneration
NfL	Axons	Increased levels after axonal injury and chronic stress exposure; reflects white matter damage	Elevated serum NfL in PTSD and trauma-exposed populations; correlates with cognitive decline and symptom chronicity	Marker of axonal injury and long-term neurobiological burden
Tau	Neuronal cytoskeleton	Stress-induced hyperphosphorylation in animal models; synaptic and microtubule instability	Mild-to-moderate elevation in PTSD; associated with memory impairment and limbic dysfunction	Indicator of cytoskeletal instability and neurodegenerative-like processes
Cytokines (IL-6, IL-18)	Microglia, astrocytes	Upregulated following stress exposure; linked to NLRP3 activation	Increased circulating levels in PTSD; correlate with symptom severity	Reflect inflammatory burden and immune–brain interaction
Oxidative stress markers (MDA, GSH)	Neuronal and glial metabolism	Elevated oxidative damage in hippocampus and PFC	Altered redox balance in PTSD patients	Support role of oxidative stress in neuroprogression

The table summarizes key biomarkers involved in astroglial activation, neuroinflammation, axonal injury, and oxidative stress in PTSD, integrating data from animal models and human studies. Abbreviations: GFAP, glial fibrillary acidic protein; S100B, S100 calcium-binding protein B; UCHL-1, ubiquitin C-terminal hydrolase L1; NfL, neurofilament light chain; BBB, blood–brain barrier; IL, interleukin; MDA, malondialdehyde; GSH, reduced glutathione; PTSD, post-traumatic stress disorder; PFC, prefrontal cortex.

**Table 3:** Comparison of biomarker profiles in PTSD, traumatic brain injury (TBI), and Alzheimer’s disease (AD).

Feature/ Biomarker	PTSD	TBI (Mild–Moderate)	AD
Primary trigger	Psychological trauma, chronic stress	Mechanical brain injury	Progressive neurodegeneration
GFAP	Chronically elevated (astroglial reactivity, BBB dysfunction)	Acutely elevated, correlates with injury severity	Moderately elevated (astrocytosis)
UCHL-1	Persistent elevation reflecting neuronal stress and proteostasis imbalance	Sharp acute increase after injury	Variable, linked to neuronal degeneration
NfL	Moderately elevated, long-lasting axonal microdamage	Strong elevation proportional to axonal injury	Markedly elevated, progressive
Tau/p-tau	Mild to moderate increase, stress-related phosphorylation	Transient increase (especially p-tau)	Strong and progressive accumulation
S100B	Elevated, reflects BBB permeability and astrocyte activation	Acutely elevated	Moderately elevated
Neuroinflammation	Chronic, low-to-moderate	Acute, intense	Chronic, progressive
BBB dysfunction	Functional, potentially reversible	Structural and functional	Progressive
Reversibility	High with treatment	Partial	Low

Table 3 compares biomarker profiles across PTSD, mild-to-moderate traumatic brain injury, and Alzheimer’s disease, highlighting differences in triggering factors, neuroinflammatory patterns, blood–brain barrier dysfunction, biomarker dynamics, and reversibility of pathological changes. Abbreviations: PTSD, post-traumatic stress disorder; TBI, traumatic brain injury; AD, Alzheimer’s disease; GFAP, glial fibrillary acidic protein; BBB, blood–brain barrier; UCHL-1, ubiquitin C-terminal hydrolase L1; NfL, neurofilament light chain; p-tau, phosphorylated tau. Evidence base: biomarker trajectories and disorder-specific patterns were synthesized from [20,21,22,23,24,83].

**Table 4:** Diagnostic and prognostic utility of combined biomarker panels in PTSD.

Biomarker Panel	Biological Domain	Clinical Application	Interpretative Value
GFAP + S100B	Astroglial activation, BBB integrity	Detection of stress-related BBB dysfunction	Indicates astrocyte reactivity and vascular permeability
UCHL-1 + NfL	Neuronal and axonal injury	Assessment of neuronal microdamage	Reflects synaptic and axonal integrity
Tau + NfL	Cytoskeletal and axonal degeneration	Evaluation of cognitive impairment risk	Links PTSD with neurodegenerative-like mechanisms
GFAP + UCHL-1	Glial–neuronal interface	Early detection of brain microinjury	High translational value (FDA-approved combination)
Biomarkers + cytokines (IL-6, IL-18)	Neuroinflammation	Symptom severity stratification	Reflects inflammatory burden
Biomarkers + oxidative stress markers (MDA, GSH)	Oxidative damage	Prognosis and treatment monitoring	Indicates redox imbalance and recovery potential
Biomarkers + psychometrics (CAPS-5, PSQI)	Bioclinical integration	Precision diagnosis and outcome prediction	Improves diagnostic accuracy and personalization

Table 4 summarizes selected combined biomarker panels integrating astroglial, neuronal, inflammatory, oxidative, and psychometric measures for PTSD. The table highlights their biological domains, clinical applications, and interpretative value, emphasizing the added diagnostic and prognostic utility of multi-marker approaches compared with single biomarkers. Abbreviations: GFAP, glial fibrillary acidic protein; S100B, S100 calcium-binding protein B; BBB, blood–brain barrier; UCHL-1, ubiquitin C-terminal hydrolase L1; NfL, neurofilament light chain; IL-6, interleukin-6; IL-18, interleukin-18; MDA, malondialdehyde; GSH, reduced glutathione; CAPS-5, Clinician-Administered PTSD Scale for DSM-5; PSQI, Pittsburgh Sleep Quality Index; FDA, U.S. Food and Drug Administration.

## Data Availability

No new data were created or analyzed in this study. Data sharing is not applicable to this article.

## Abbreviations

The following abbreviations are used in this manuscript:

AD	Alzheimer’s disease
AMPA	α-amino-3-hydroxy-5-methyl-4-isoxazolepropionic acid
ASC	apoptosis-associated speck-like protein containing a caspase recruitment domain
ATP	adenosine triphosphate
BBB	blood–brain barrier
BDI	Beck Depression Inventory
BDNF	brain-derived neurotrophic factor
CAPS-5	Clinician-Administered PTSD Scale for DSM-5
CBT	cognitive behavioral therapy
CD11b	cluster of differentiation 11b
CDK5	cyclin-dependent kinase 5
CECS	Courtauld Emotional Control Scale
CNS	central nervous system
CSF	cerebrospinal fluid
CT	computed tomography
CTE	chronic traumatic encephalopathy
CX3CL1	C-X3-C motif chemokine ligand 1
CX3CR1	C-X3-C motif chemokine receptor 1
CXCL10	C-X-C motif chemokine ligand 10
DAMPs	danger-associated molecular patterns
DNN	deep neural networks
DSM-5	Diagnostic and Statistical Manual of Mental Disorders, Fifth Edition
DTI	diffusion tensor imaging
EAAT2	excitatory amino acid transporter 2
EMDR	eye movement desensitization and reprocessing
ER	endoplasmic reticulum
FDA	U.S. Food and Drug Administration
fMRI	functional magnetic resonance imaging
GABA	gamma-aminobutyric acid
GFAP	glial fibrillary acidic protein
GSH	reduced glutathione
GSK-3β	glycogen synthase kinase 3 beta
HPA	hypothalamic–pituitary–adrenal
ICAM-1	intercellular adhesion molecule 1
IGF-1	insulin-like growth factor 1
IL-1β	interleukin-1 beta
IL-6	interleukin-6
IL-18	interleukin-18
IRE1–XBP1	inositol-requiring enzyme 1–X-box binding protein 1
JNK	c-Jun N-terminal kinase
LIME	Local Interpretable Model-agnostic Explanations
LTP	long-term potentiation
MAPK	mitogen-activated protein kinase
MCC950	CP-456, 773
MCP-1	monocyte chemoattractant protein-1
MDA	malondialdehyde
MFN2	mitofusin-2
ML	machine learning
MMP-2	matrix metalloproteinase-2
MMP-9	matrix metalloproteinase-9
MRI	magnetic resonance imaging
MS	multiple sclerosis
NAA	N-acetylaspartate
NF-κB	nuclear factor kappa B
NfL	neurofilament light chain;
NLRP3	NOD-like receptor family pyrin domain-containing 3
NMDA	N-methyl-D-aspartate
NVU	neurovascular unit
PAMPs	pathogen-associated molecular patterns
PD	Parkinson’s disease
PERK	protein kinase RNA-like endoplasmic reticulum kinase
PET	positron emission tomography
PET-TSPO	positron emission tomography targeting the 18 kDa translocator protein
PKA	protein kinase A
PPAR-γ	peroxisome proliferator-activated receptor gamma
PSD-95	postsynaptic density protein 95
PSQI	Pittsburgh Sleep Quality Index
PTSD	post-traumatic stress disorder
RAGE	receptor for advanced glycation end products
RNS	reactive nitrogen species
ROS	reactive oxygen species
SHAP	SHapley Additive exPlanations
SNRI	serotonin–norepinephrine reuptake inhibitor
SSRI	selective serotonin reuptake inhibitor
SVM	support vector machine
S100B	S100 calcium-binding protein B
TBI	traumatic brain injury
TJs	tight junctions
TLR2	Toll-like receptor 2
TLR4	Toll-like receptor 4
TNF-α	tumor necrosis factor alpha
TNFR1	tumor necrosis factor receptor 1
TSPO	translocator protein
TXNIP	thioredoxin-interacting protein
UCHL-1	ubiquitin C-terminal hydrolase L1
VCAM-1	vascular cell adhesion molecule 1
ZO-1	zonula occludens-1
